# Short-term outcomes of laparoscopic local resection for gastric submucosal tumors: a single-center experience of 266 patients

**DOI:** 10.1186/s12893-017-0231-0

**Published:** 2017-04-04

**Authors:** Ke Chen, Yu Pan, Shu-ting Zhai, Jun-hai Pan, Wei-hua Yu, Ding-wei Chen, Jia-fei Yan, Xian-fa Wang

**Affiliations:** Department of Gastrointestinal Surgery, Sir Run Run Shaw Hospital, School of Medicine, Zhejiang University, 3 East Qingchun Road, Hangzhou, 310016 Zhejiang Province China

**Keywords:** Laparoscopy, Gastrectomy, Submucosal tumors, Complications

## Abstract

**Background:**

Laparoscopic resections for submucosal tumors (SMTs) of the stomach have been developed rapidly over the past decade. Several types of laparoscopic methods for gastric SMTs have been created. We assessed the short-term outcomes of two commonly used types of laparoscopic local resection (LLR) for gastric SMTs and reported our findings.

**Methods:**

We retrospectively analyzed the clinicopathological results of 266 patients with gastric SMTs whom underwent LLR between January 2006 and September 2016. 228 of these underwent laparoscopic exogastric wedge resection (LEWR), the remaining 38 patients with the tumors near the esophagogastric junction (EGJ) or antrum underwent laparoscopic transgastric resection (LTR).

**Results:**

All the patients underwent laparoscopic resections successfully. The mean operation times of LEWR and LTR were 90.2 ± 37.2 min and 101.7 ± 38.5 min respectively. The postoperative length of hospital stays for LEWR and LTR were 5.1 ± 2.1 days and 5.3 ± 1.7 days respectively. There was a low complication rate (4.4%) and zero mortality in our series.

**Conclusion:**

ELWR is technically feasible therapy of gastric SMTs. LTR is secure and effective for gastric intraluminal SMTs located near the EGJ or antrum.

## Background

Submucosal tumors (SMTs) of the stomach are defined as tumors located beneath the gastric mucosa and include gastrointestinal stromal tumors (GISTs), schwannomas, leiomyomas, malignant lymphomas, lymphangiomas, lipomas, hemangiomas, heterotopic pancreas, *et al.* [1]. We can always perceive gastric SMTs accidently, which make up 2% of whole gastric neoplasms [2]. A broad clinical spectrum was shown from benign to malignant. It seems tough to deal with diagnosis of the tumors before the operation and evaluation of the extent of latent malignancy. Moreover, even a benign tumor can cause a variety of complications such obstruction and bleeding. Therefore, surgical excision of the lesions remains the first choice. Achieving a disease-free margin to complete the partial excision of the tumor is favored and lymphadenectomy is not usually needed, because nearly 80% of gastric SMTs are GISTs, whose periodicity of lymph node metastasis is low [3, 4].

Laparoscopic resection, which has been regarded as the most appropriate treatment, not only offers minimal normal tissue loss and maintains gastrointestinal continuity, but is also characterized by minimally invasive to the patient. Exogastric laparoscopic wedge resection (ELWR) is the most prevailing laparoscopic local resection (LLR). However, as to neoplasms located at cardia, especially near the esophagogastric junction (EGJ), or lay to antrum, LEWR increases the risk of generating stenosis or deformity in the gastric inlet or outlet [5]. Based on our extensive laparoscopic gastrectomy (LG) [6–10], we developed laparoscopic transgastric resection (LTR) for tumors located at cardia or antrum to avoid a total, proximal or distal gastrectomy. We herein give notice to our results from these two types of laparoscopic resection methods and also an assessment of the postoperative surgical outcomes to evaluate the feasibility and safety of those procedures.

## Methods

### Patients

At the Department of General Surgery, Sir Run Run Shaw Hospital, 266 patients suffered LLR for probable gastric SMTs. In order to assess the site, dimension, and development mode of the tumor, all of patients experienced preoperative process, including gastroscopy, endoscopic ultrasound (EUS), and abdominal computed tomography (CT). Some patients with enormous tumors were diagnosed before the operation, whose tumors may have intruded on neighboring organs, including various organs because of metastatic disease, or suffered an emergency surgery due to acute upper gastrointestinal bleeding. These people were not taken account into the research. Written consent was obtained from every patient prior to enrollment in the study, which was confirmed by the Zhejiang University’s Ethics Committee.

### Data collection

People analyzed retrospectively and kept track of demographic message, surgical procedures, pathologic message, clinical presentation, and the process for these patients after operation. Pathologic features of GIST patients were studied, including tumor size, location, mitotic rate and Fletcher classification [11]. On the basis of the Risk Assessment Classification presented by Fletcher and colleagues, the GISTs were quadripartite. [10] (National Institutes of Health [NIH] consensus criteria). A skilled pathologist counted mitotic figures for total specimen in 50 high-power fields (HPFs), which were chosen at random. We also analyzed surgical outcomes involved with the loss of blood, the time of operation, and complications after operation *et al*.

### Surgical procedure

**Fig. 1:**
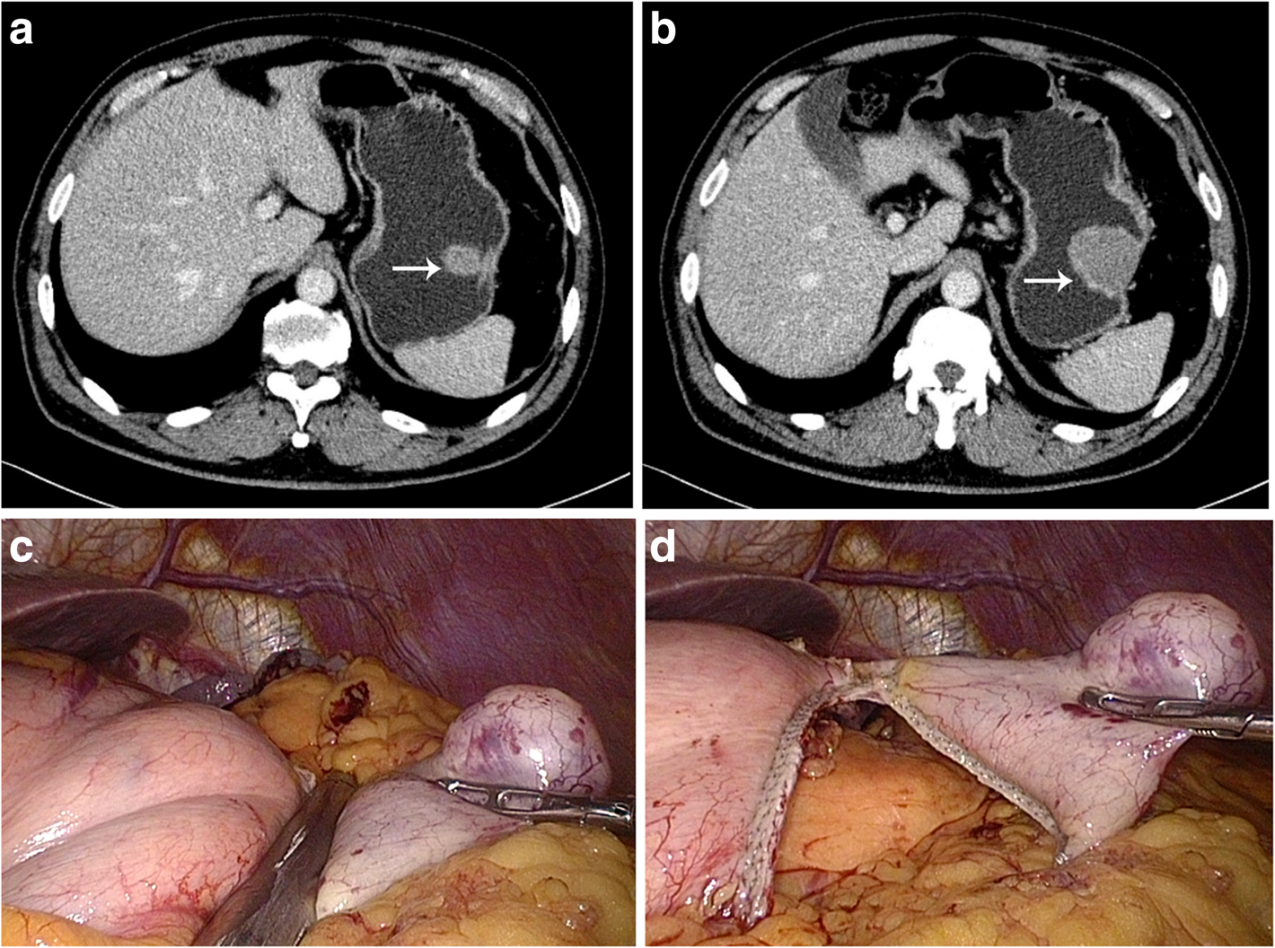
Resection of tumor in anterior wall of gastric body. (**a**) Image of the tumor from abdominal CT scan. (*white arrow*). (**b**) Image of the tumor from abdominal CT scan. (*white arrow*). (**c**) Resect the wall included the gastric SMTs using linear stapler. (**d**) Fire the anastomat and complete the resection

**Fig. 2:**
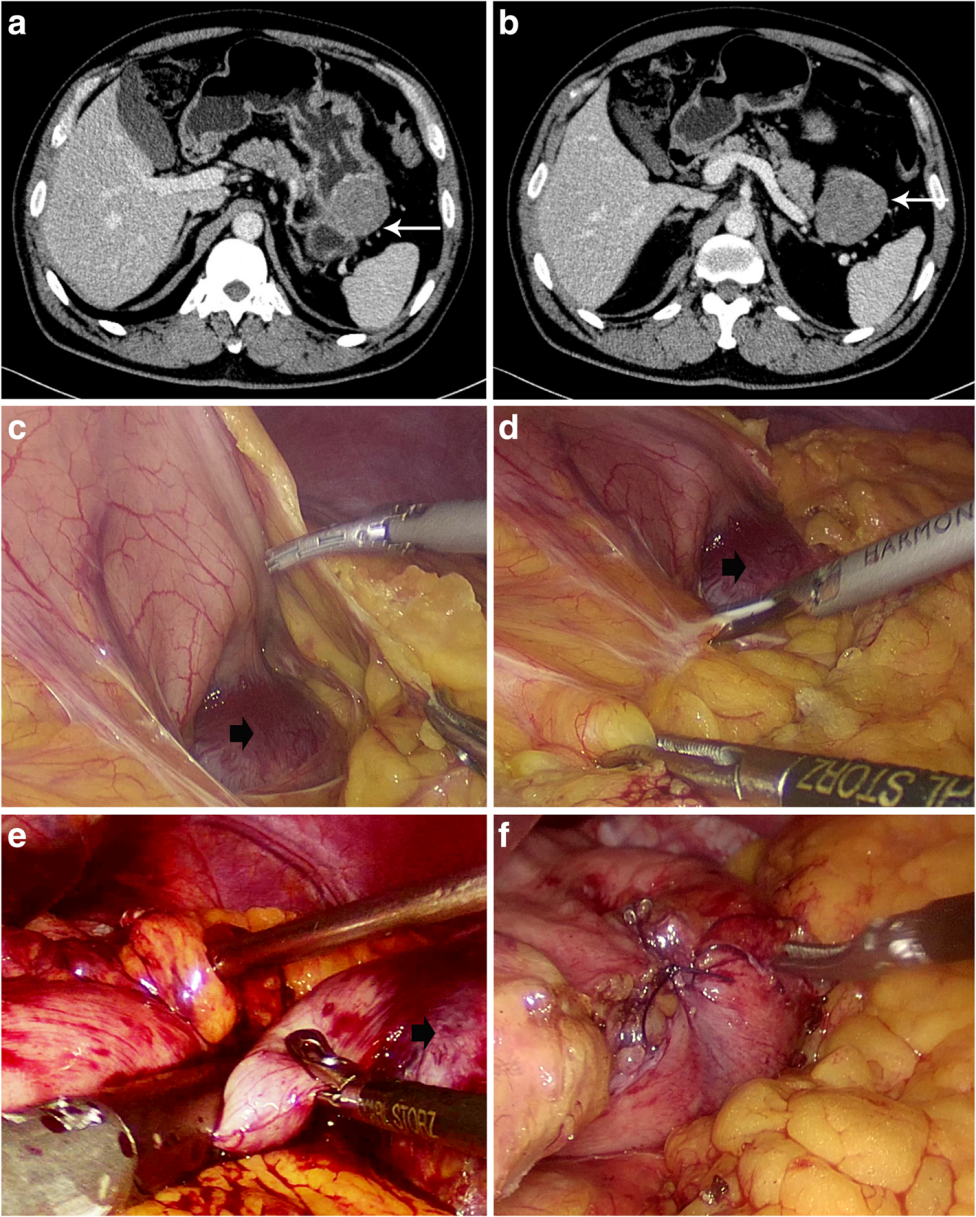
Resection of tumor in posterior wall of gastric body. (**a**) Image of the tumor from abdominal CT scan. (*white arrow*). (**b**) Image of the tumor from abdominal CT scan. (*white arrow*). (**c**) Open the greater omentum to splenic hilum. (*black arrow*). (**d**) Dissect the pancreatic stomach plica to expose the tumor. (*black arrow*). (**e**) Resect the wall included the gastric SMTs using linear stapler. (**f**) Complete the resection

**Fig. 3:**
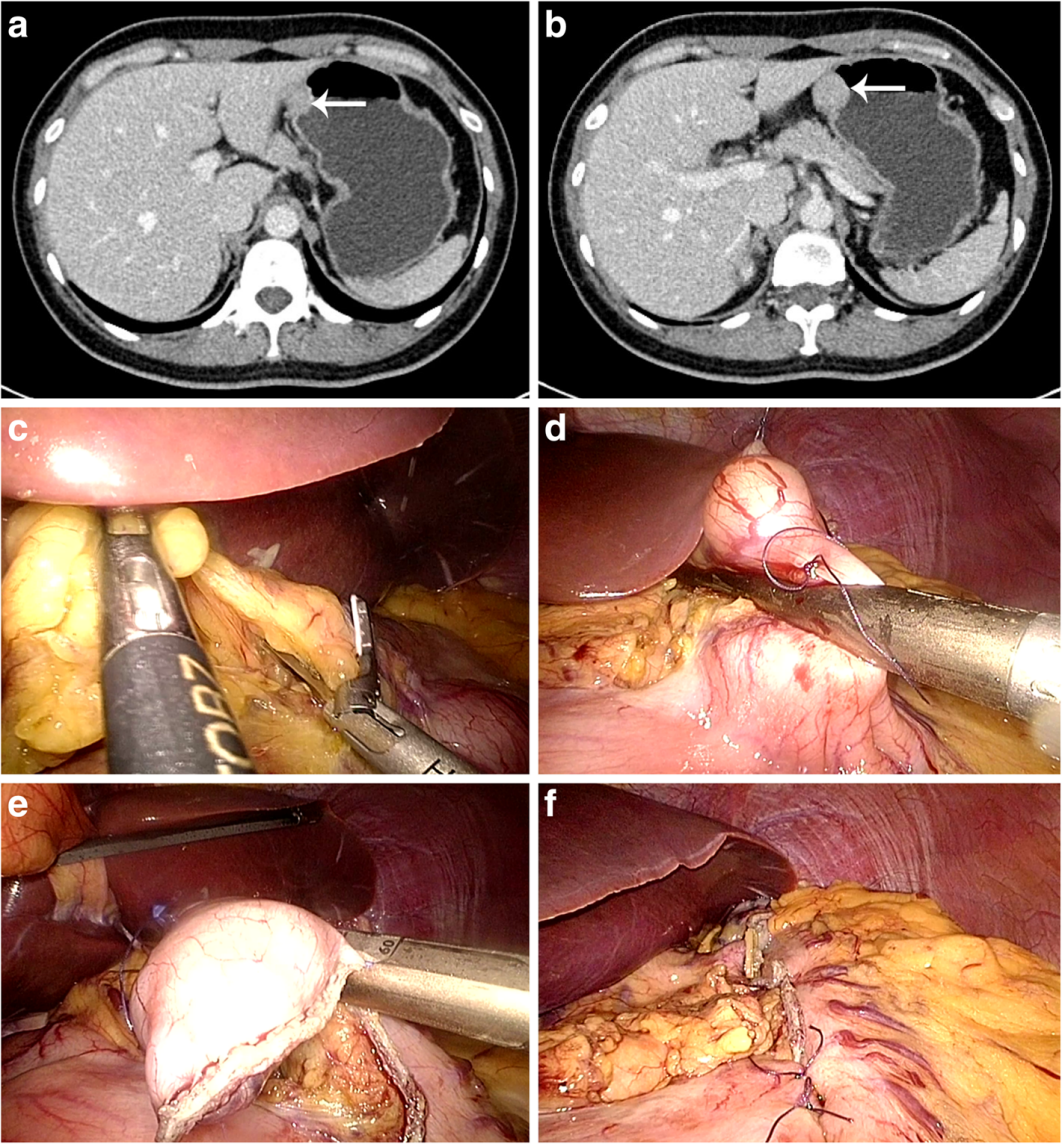
Resection of tumor in lesser curvature. (**a**) Image of the tumor from abdominal CT scan. (*white arrow*). (**b**) Image of the tumor from abdominal CT scan. (*white arrow*). (**c**) Explore the hepatogastric ligament. (**d**) Resect the wall included the gastric SMTs using linear stapler. (**e**) Complete the resection using another stapler. (**f**) Reinforce the resection using several sutures

**Fig. 4:**
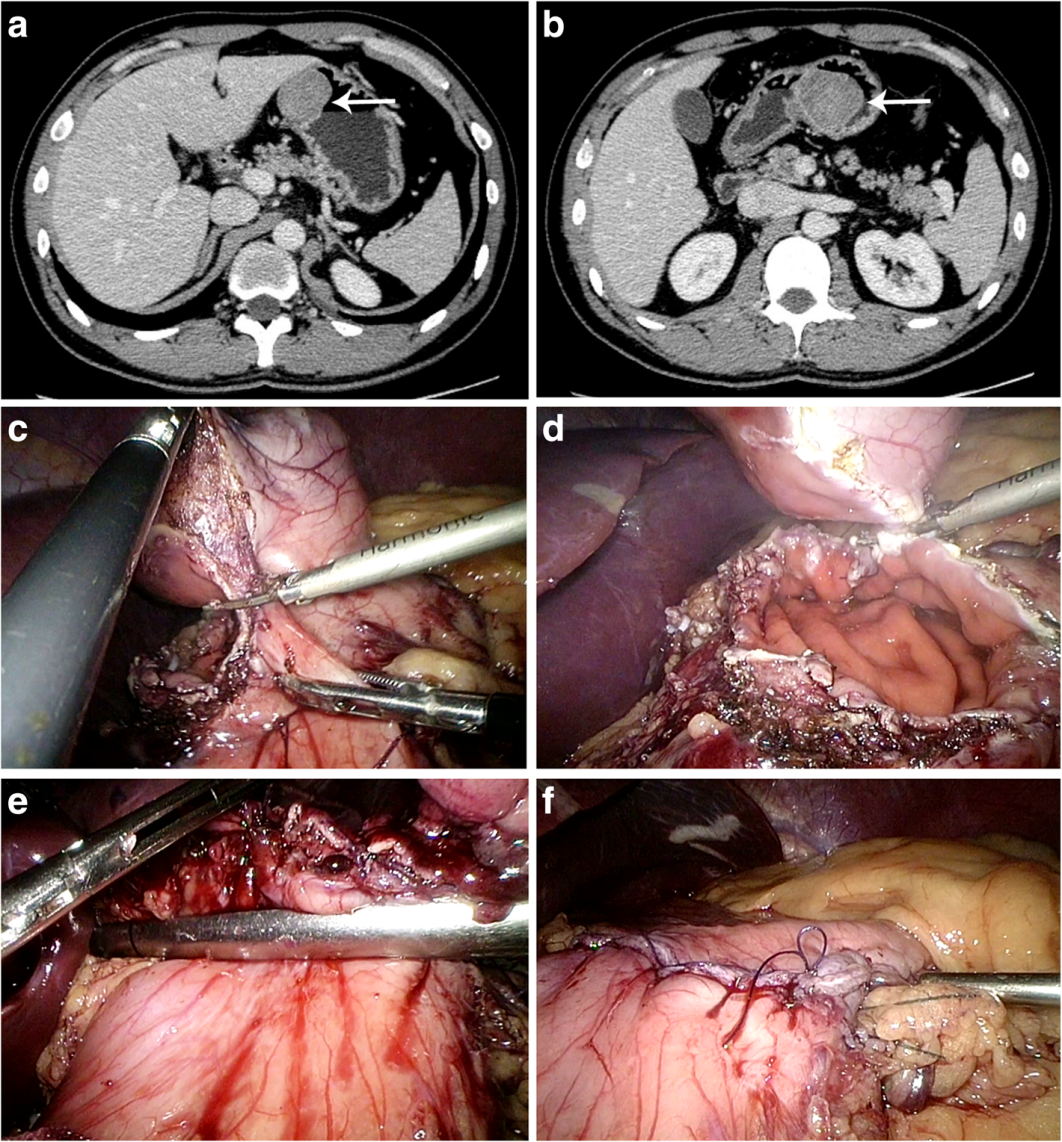
Resection of tumor using ultrasonic coagulating shears. (**a**) Image of the tumor from abdominal CT scan. (*white arrow*). (**b**) Image of the tumor from abdominal CT scan. (*white arrow*). (**c**) Resect along the tumor using ultrasonic coagulating shears. (**d**) Resect the tumor. (**e**) Close the opening using endoscopic linear staplers. (**f**) Reinforce the resection using several sutures

The former described approach was utilized for site and trocar place [6]. We built five trocars in a V-shape setting. In order to eliminate tumor spread and transfer, we operate an entire laparoscopic abdominal examination before the resection. Gastroscopy was taken advantage to assess tumor positioning during the operation if necessary. Tumor positions were first made clear by laparoscopic handing when using LEWR. Before excision, we always mobilize the tumor as follows: Tumor in anterior wall of the gastric body was excised directly using ultrasonic coagulating shears or endoscopic linear staples (Fig. 1). It will be effortless to redeploy the tumor by taking advantage of ultrasonic coagulating shears, where the greater omentum incision was originated from the middle-inferior pole of the spleen to the greater outer winding of the vessel of the gastric omentum. Moreover, in order to redeploy the tumor, the hepatogastric ligament was anatomized. They anatomized the gastrocolic and gastrosplenic ligaments, and then held up the stomach to make the tumor clear for tumors which were lay on the posterior wall (Figs. 2 and 3). In order to redeploy the fundus and make the tumor clear, they also anatomized gastrocolic and gastrosplenic ligament as well as left gastroepiploic vessels and short gastric vessels for tumors which were in fundus. Utilizing ultrasonic coagulating shears or endoscopic staples with at lowest 1–2 cm surgical margin, we resected the tumor. Based on the measurement of the tumor, each excision needed 2 to 3 staples. If the tumor was relatively large, we recommend resecting the tumor using ultrasonic coagulating shears. In order to hold the corners, several sutures were utilized as long stay sutures, and the endoscopic linear staplers played the role of concluding the start. The defect from the stapler line was reinforced using laparoscopic manual sutures to avoid bleeding or leakage (Fig. 4).

**Fig. 5:**
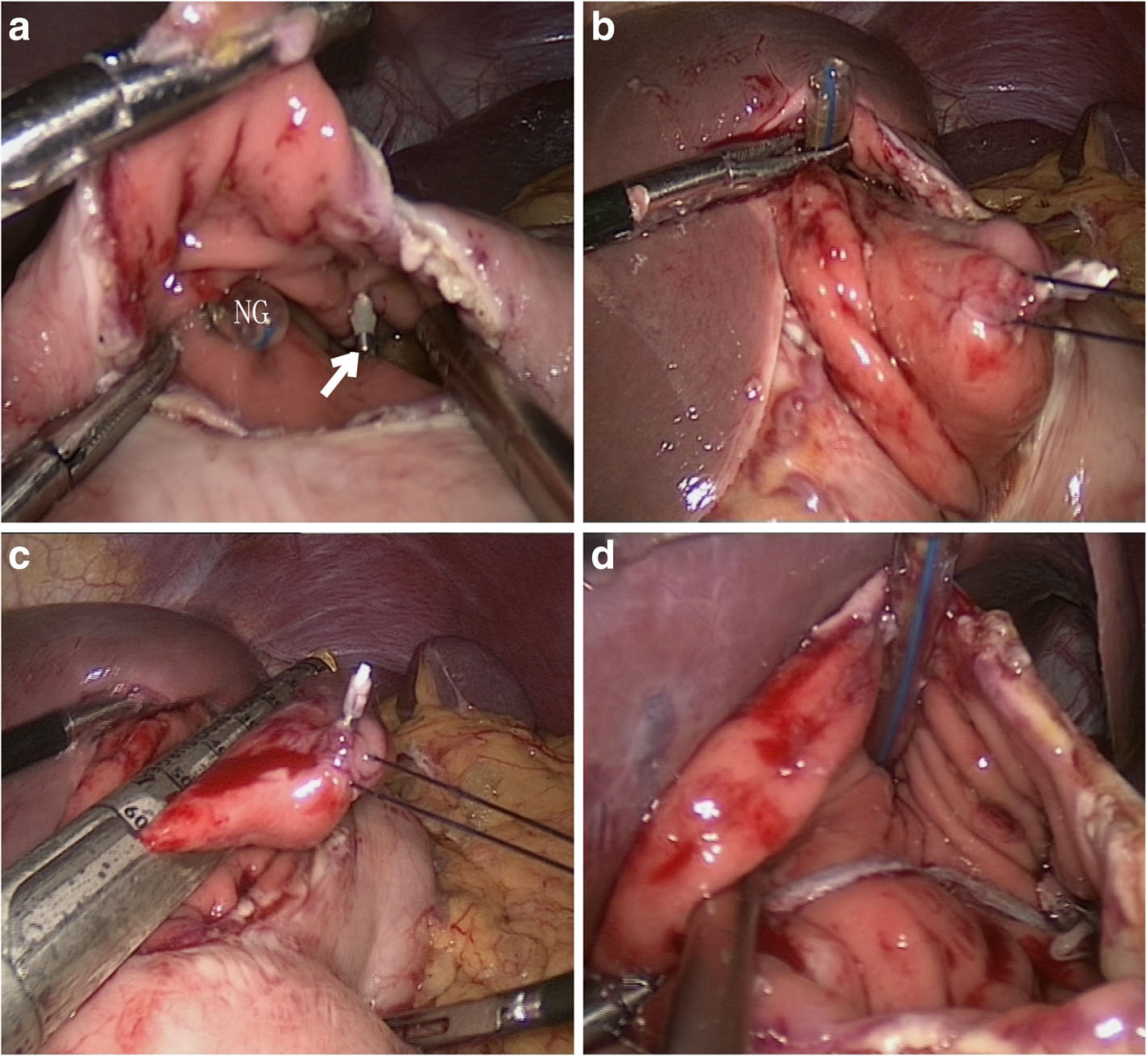
laparoscopic transgastric resection of gastric SMTs located near the EGJ. (**a**) A gastrotomy was performed at the anterior wall of the proximal stomach and the SMTs marked with the titanium clip and mucosa of EGJ were directly observed from the openings. (arrow: SMTs marked with the titanium clip; NG: nasogastric tube). (**b**) Evert the tumor from the gastrotomy by a stay suture. (**c**) Stapled resection of the tumor. (**d**) Complete the transgastric resection

We used LTR for tumors located at cardia, near EGJ or the antrum, especially those with intraluminal growth, to avoid deformity or stenosis in the gastric inlet or outlet. If the tumor was located in the cardia, the process was originated from distributing the gastrocolic ligament up to the spleen or the duodenum degree. In order to come near the EGJ, the hepatogastric or hepatoduodenal ligament was opened. We used ultrasonic coagulating shears to make a perpendicular gastrotomy cut on the anterior wall of the stomach at the possible site of the tumor after redeployment of the EGJ. It was effortless to observe the SMTs marked with the titanium clip and the mucosa directly from the opening (Fig. 5a). Using a stay suture, we upturned the full-thickness gastric wall from this gastrotomy in the lesion area (Fig. 5b). In order to excising transgastricly, we used several endoscopic linear staples to excise the tumor with the entire layer of gastric wall (Fig. 5c.d). Then, the utilizing of endoscopic linear staples longitudinally or laparoscopic manual sutures brought an end to the gastrotomy. The tumor was retrieved from the umbilical wound and placed in a specimen bag.

If the imageological examinations or intraoperative findings showed the tumor was near the pylorus. Resection commenced with dissection of the appropriate section of the greater omentum with ultrasonic coagulating shears. The short gastric vessels near the tumor were then transected to a height dependent on the level of transection. The right gastric artery was divided and cut. The left lobe of the liver was retracted upward, while the stomach was stretched downward to expose the lesser omentum. At this stage, dissection was continued at the side with the least curvature of the stomach. After mobilization of the pylorus, the excision was similar to those near EGJ.

## Results

### Patient characteristics

The clinical characteristics of the 266 patients whom underwent laparoscopic surgical resection of gastric SMTs are summarized in Table 1. The 266 patients included 91 males and 175 females. The mean age was 57.8 years (range 22 to 81 years). The mean body mass index (BMI) was 23.4 kg/m^2^ (range: 13.6 to 31.3). Of these patients, 228 underwent LEWR and 38 patients underwent LTR. According to the pathological reports, 229 patients were diagnosed with GIST. The remaining patients were diagnosed with submucosal tumors other than GIST, such as schwannomas (15 patients), ectopic pancreas (4 patients), leiomyoma (14 patients), lipomas (2 patients) and plasmacytoma (2 patients).

**Table 1:** Clinical characteristics

Variable	LEWR (*n* = 228)	LTR (*n* = 38)
Gender (male/female)	78/150	13/25
Age (years)	58.7 ± 11.8	52.4 ± 10.3
BMI (kg/m2)	23.4 ± 3.6	23.5 ± 2.7
ASA classification (I/II/III)	113/108/7	19/15/4
Comorbidities (yes)	104	16
Hypertension	77	9
Diabetes mellitus	23	7
Cardiovascular	13	2
Pulmonary	6	3
Pathology		
GIST	203	26
Schwannoma	12	3
Ectopic pancreas	3	1
Leiomyoma	7	7
Lipomas	1	1
Plasmacytoma	2	0

*Abbreviation*: *BMI* body mass index, *ASA* American Society of Anesthesiologists

Data are means ± standard deviations or number

### Pathologic features of GIST patients

As shown in Table 2, among the patients with GISTs, 203 suffered from LEWR and 26 suffered from LTR. The mean tumor size of LEWR group was 3.6 ± 2.5 cm, and that of LTR group was 2.1 ± 1.3 cm. On the basis of pathological reports, 202 patients were reported to have a mitotic rate of < 5 per 50HPF, 18 patients were reported to have a mitotic rate of 5 ~ 10 per 50HPF and 8 patients were reported to have a mitotic rate of > 10 per 50HPF. Adopting the standard of Fletcher classification, our patients were assigned to four groups: very low risk (68 patients), low risk (111 patients), intermediate risk (36 patients) and high risk (14 patients).

**Table 2:** Pathologic features of GIST patients

Variable	LEWR (*n* = 203)	LTR (*n* = 26)
Tumor size (cm)	3.6 ± 2.5	2.1 ± 1.3
Mitotic rate (per 50 HPF)		
<5	180	22
5 ~ 10	15	3
>10	8	0
Fletcher classification		
Very low risk	55	13
Low risk	100	11
Intermediate risk	34	2
High risk	14	0

### Surgical and postoperative outcomes

As shown in Table 3, the mean operation time of LTR was 101.7 ± 38.5 min and was 90.2 ± 37.2 min in LEWR group. The mean blood loss of LEWR and LTR were 50.4 ± 51.6 mL and 42.2 ± 30.2 mL respectively. The postoperative hospital stay of LEWR and LTR were 5.1 ± 2.1 days and 5.3 ± 1.7 days respectively. In the LEWR group, 1 patient suffered from intraluminal bleeding, 4 patients suffered from delayed gastric emptying and 2 patients suffered from pulmonary infections. This group had a complication rate of 3.07%. Two patients (7.14%) developed intraluminal bleeding and one patient developed a pulmonary infection in the LTR group. There were no incidences of conversion to open surgery during the operation. All patients with complications were cured with a conservation treatment. There was no perioperative mortality in our series.

**Table 3:** Surgical outcomes of 266 patients

Variable	LEWR (*n* = 228)	LTR (*n* =38 )
Operation time (min)	90.2 ± 37.2	101.7 ± 38.5
Blood loss (mL)	50.4 ± 51.6	42.2 ± 30.2
First flatus (day)	2.4 ± 1.1	2.2 ± 0.9
Oral intake (days)	3.2 ± 1.1	3.0 ± 1.0
Postoperative hospital stay (days)	5.1 ± 2.1	5.3 ± 1.7
Postoperative complications		
Intraluminal bleeding	1	2
Delayed gastric emptying	4	
Abdominal abscess		
Pulmonary infection	2	1

## Discussion

Gastric SMTs are potentially life-threatening tumors. Although the majority of SMTs are benign, some have the potential to become malignant. GISTs make up a large portion of SMTs. It has been reported that the pathogenesis of GIST is closely related to mutations at c-Kit proto-oncogene, and GISTs that do not have c-Kit mutations might be correlated with gain-of-function mutations of platelet-derived growth factor-a (PDGFRa) [12]. Secondary to these findings, imatinib mesylate (Gleevec®) therapy, which can inhibit the intracellular kinase activities of CD117 and PDGFRa, has been manifested to increase the overall tumor control rate of GIST by 85%. It has been report that Gleevec® is the standard treatment for unresectable or metastatic GIST in many countries [13]. However, resection remains the first option for primary GIST. On the one hand, it can establish the diagnosis; on the other hand, it may be curative. SMTs that are not obviously benign should be excised as assumed GISTs. Patients with a diagnosis for GISTs are mainly treated by laparoscopic local resection [14].

Lukaszczyk and colleagues reported in 1992 the use of laparoscopy for gastric GIST resection in a patient [15]. Since then, there have been multiple small series using the laparoscopic approach for these kinds of tumors. In this study, LLR was successfully performed within an acceptable operation time. It is lowest of the average blood loss and morbidity rate, and mortality hardly happened. All patients started oral feeding earlier, the average oral intake day is 3.2 days (range: 2 to 6 days) after the operation. Hospital stays were also short and acceptable. Pathologic examination of the surgical specimens showed that all surgical margins were microscopically tumor free (R0 resection). Although there are no randomized studies comparing laparoscopic versus open approach in the management of gastric SMTs, several retrospective studies have shown the advantages of the laparoscopic approach in treating gastric SMTs, with similar disease free survival, mortality and oncologic outcomes comparable with the open approach [16–24]. It is not only the technical potential of laparoscopic excision, but also its effectiveness facilitated by the data.

LLR can be distributed into ELWR or LTR according to the development mode, tumor measurement, and site. ELWR is the most ordinary method and the first thought for gastric SMTs. We can use the laparoscopic vision or the haptic retroaction of the laparoscopic tools to locate lesions straightly for the tumors which were located in the anterior wall along the greater curvature of the stomach. Considering the remarkable superfluity and mobility of the stomach in these sites, a laparoscopically stapled gastric wedge excision often is always greatly potential. With a project for a stapled wedge excision, it is easy to come near laparoscopically for tumors in this site. In order to avoid cardiac stricture for tumors located in the fundus, the left gastroepiploic artery should be transected. The gastrophrenic ligament and gastrosplenic ligament should be dissected for mobility of the stomach and then making sure there is enough distance between the cutting line and the left side of the cardia when applying the second staple. Otherwise it would cause stricture due to the Endo-GIA position being too close to the cardia. Because the stomach where tumors along the lesser curvature are short of redundancy, and the lesser curvature is restricted in length, so it is tough to treat laparoscopically. To make the posterior wall of the stomach clear, we are supposed to do dissection to part the stomach from the greater omentum. Then, the lesser curvature was everted and the tumor was removed. The vagus nerve branch (Latarjet nerves) and blood vessels in the lesser omentum should be protected.

For the reason that ELWR has the chance of stenosis or deformity conducing to excessive excision of the normal gastric wall, it is difficult to apply to tumors which are located close to the gastric inlet or outlet [5]. In our series, there seemed to be 65 cases with tumors which were located close to the esophagogastric junction or the pylorus which were thought improper to undergo ELWR by laparoscopic stapling. We had better utilize ultrasonic coagulating shears with the excision margin paralleling the round edge of the tumor to reduce the fine tissue loss and to hold back luminal narrowing to operate manual excision, on condition that the tumor has exogastric development and is located on the anterior wall. Then, the laparoscopic intracorporeal hand-sewn method was used to close the incision. It is advisable to use the laparoscopic transgastric method for the excision of an intraluminal tumor which is located at the posterior wall of the stomach, which offered straight observation of the lesion and inner stomach, and brings greater command of the surgical margin. [25, 26]. The outcomes of our retrospective research proved that the process was secure and effective. Two cases had postoperative gastric intraluminal bleeding, but this was cured with a conservation treatment. In our experience, the exact site of tumors is crux of this method, which is using endoscopic before the operation, marking with titanium clips, intact redeployment of the EGJ or pylorus before excision, and step cutting transversely along the foundation of the tumor. However, restrictions related to attached bleeding venture and intraperitioneal contanmination by seeping gatric juices are common.[26, 27]. The endoscopic linear staple resection line of the stomach wall is weak and leakage and bleeding occur easily. Therefore, intracorporeal hand-sewn was used in our center to reinforce this area. Intraperitioneal contamination with gastric juice seemed to be a dominating problem of this technique, as the gastric cavity requires opening temporarily. Hence, it is necessary to operate abundant decompression of the stomach before gastrotomy and comprehensive irrigation of the operating area after closing the gastrotomy for preventing abdominal or wound infection.

There is another technique designed to resect tumors located near the EGJ or pylorus is laparoscopic intragastric resection [26]. This technique involves a difficult procedure to set up the view before resection. By blowing up a balloon sticked on the trocars, the gastric wall is supposed to be attached to the abdominal wall after inserting several trocars into the gastric lumen through the gastric wall. This method not only offers an abundant operative area, but also generates less deformity of the EGJ compared with an extragastric approach. However, the need of specific ballon-type ports and the trouble originated from inserting the ports into stomach restricted its feasibility. In addition, if a tumor is larger than 4 cm, the intragastric resection is inapposite, because it is difficult to retract the large specimen orally. We did not use this method in our center.

If a relatively large tumor is located at EGJ and antrum, the surgeons must consider the problems incurred by LLR that include the possibility of stenosis and deforming the gastric inlet or outlet. For tumors located at antrum or large tumors in the lower stomach close to antrum, we recommended laparoscopic distal gastrectomy (LDG). For tumors at EGJ or large tumors in the middle or upper body we recommend laparoscopic total gastrectomy (LTG) instead of laparoscopy proximal gastrectomy (LPG) due to the relatively lower rate of reflux esophagitis. Another tip, observation by flexible endoscope during the resection of the GIST is recommended to avoid gastric inlet or outlet narrowing. During endoscope examination, we looped the pylorus with a silk band to stop gas from entering the intestine, which would interfere with vision and subsequent manipulation.

Several limitations of this study warrant mention and require special attentions in the interpretation. First, because it was a retrospective study performed at a single institution, case selection was inevitably affected by bias. Second, the uneven surgical skills of the different surgeons might result in flaws of the study. Third, it’s a one-arm study and long-term outcomes were not evaluated because of the short observation period. Therefore, randomized controlled trials or prospective comparative studies with long-term follow-up are necessary to adequately evaluate the status of LLR for gastric SMTs.

## Conclusion

In conclusion, for the reason that the process provides reasonable morbidity and well-pleasing short-term outcomes, it seems that ELWR is technically practicable for the therapy of gastric SMTs. For gastric intraluminal SMTs which are located close to the EGJ or pylorus, LTR is easy, secure, and efficient. When LLR are inappropriate for bulky tumors located at EGJ or antrum, LDG or LTG could be used to avoid stenosis.

## Abbreviations

CT: Computed tomography
EGJ: Esophagogastric junction
EUS: Endoscopic ultrasound
GISTs: Gastrointestinal stromal tumors
HPFs: High-power fields
LDG: Laparoscopic distal gastrectomy
LEWR: Laparoscopic exogastric wedge resection
LG: Laparoscopic gastrectomy
LLR: Laparoscopic local resection
LPG: Laparoscopy proximal gastrectomy
LTG: laparoscopic total gastrectomy
LTR: Laparoscopic transgastric resection
NIH: National Institutes of Health
SMTs: Gastric submucosal tumors
